# Interobserver variability studies in diagnostic imaging: a methodological systematic review

**DOI:** 10.1259/bjr.20220972

**Published:** 2023-06-29

**Authors:** Laura Quinn, Konstantinos Tryposkiadis, Jon Deeks, Henrica C.W. De Vet, Sue Mallett, Lidwine B. Mokkink, Yemisi Takwoingi, Sian Taylor-Phillips, Alice Sitch

**Affiliations:** 1 Test Evaluation Research Group, Institute of Applied Health Research, University of Birmingham, Birmingham, United Kingdom; 2 NIHR Birmingham Biomedical Research Centre, University Hospitals Birmingham NHS Foundation Trust and University of Birmingham, Birmingham, United Kingdom; 3 Department of Epidemiology and Data Science, Amsterdam UMC, Vrije Universiteit Amsterdam, Amsterdam, The Netherlands; 4 Amsterdam Public Health Research Institute, Amsterdam, The Netherlands; 5 Centre for Medical Imaging, University College London, London, United Kingdom; 6 Division of Health Sciences, Warwick Medical School, University of Warwick, Coventry, United Kingdom

## Abstract

**Objectives:**

To review the methodology of interobserver variability studies; including current practice and quality of conducting and reporting studies.

**Methods:**

Interobserver variability studies between January 2019 and January 2020 were included; extracted data comprised of study characteristics, populations, variability measures, key results, and conclusions. Risk of bias was assessed using the COSMIN tool for assessing reliability and measurement error.

**Results:**

Seventy-nine full-text studies were included covering various imaging tests and clinical areas. The median number of patients was 47 (IQR:23–88), and observers were 4 (IQR:2–7), with sample size justified in 12 (15%) studies. Most studies used static images (*n* = 75, 95%), where all observers interpreted images for all patients (*n* = 67, 85%). Intraclass correlation coefficients (ICC) (*n* = 41, 52%), Kappa (κ) statistics (*n* = 31, 39%) and percentage agreement (*n* = 15, 19%) were most commonly used. Interpretation of variability estimates often did not correspond with study conclusions. The COSMIN risk of bias tool gave a very good/adequate rating for 52 studies (66%) including any studies that used variability measures listed in the tool. For studies using static images, some study design standards were not applicable and did not contribute to the overall rating.

**Conclusions:**

Interobserver variability studies have diverse study designs and methods, the impact of which requires further evaluation. Sample size for patients and observers was often small without justification. Most studies report ICC and κ values, which did not always coincide with the study conclusion. High ratings were assigned to many studies using the COSMIN risk of bias tool, with certain standards scored ‘not applicable’ when static images were used.

**Advances in knowledge:**

The sample size for both patients and observers was often small without justification.

For most studies, observers interpreted static images and did not evaluate the process of acquiring the imaging test, meaning it was not possible to assess many COSMIN risk of bias standards for studies with this design.

Most studies reported intraclass correlation coefficient and κ statistics; study conclusions often did not correspond with results.

## Introduction

Obtaining an accurate medical diagnosis is fundamental to receiving the correct treatment. Medical imaging is an essential part of diagnostic pathways, with estimations that 3–5% of all diagnoses have errors, meaning up to 40 million diagnostic errors could be made yearly worldwide.^1^ Diagnostic errors have the potential to lead to significant patient harm.^2^ Having reliable diagnostic imaging tests in clinical practice means that patients can benefit from the best and most appropriate treatments. Removing the less reliable tests can reduce diagnostic errors, reduce resource waste and provide better patient care.

Studies evaluating diagnostic imaging tests usually consider the accuracy of a test but do not always measure interobserver variability, which is another important aspect of evaluating diagnostic test performance. Interobserver variability is the variability of test results when different observers perform the same test on the same patient.^3^ When using imaging tests compared to other types of tests (*e.g.,* laboratory-based tests), there is additional variability caused by different observers interpreting the images.^4^ In this review, interobserver variability will be used as an umbrella term to cover both reliability and measurement error, which are two related but distinct measurement properties.^5^ Reliability is defined as the ratio of variability in measurement interpretations between participants to the total variability of all measurements in a sample, whereas measurement error or agreement refers to the degree to which interpretations are identical.^6^

In 2011, the Guidelines for Reporting Reliability and Agreement Studies (GRRAS)^6^ were proposed to improve the reporting of studies. A systematic review focusing on the reporting of interobserver variability in imaging was also conducted.^7^ Both the guidelines and systematic review identified poor reporting and presented a limited snapshot of the design and methods used so no comments could be made on the quality of conducting the interobserver variability studies. A newly developed Consensus-based Standards for the selection of health Measurement Instruments (COSMIN) risk of bias tool for assessing reliability and measurement error (further called ‘the COSMIN tool’) was used to assess the quality of studies.^8^

The aim of this methodological review is to provide an overview of current practice, including the quality of reporting, and the quality of conducting interobserver variability studies. The review will mainly focus on study design and statistical methods to identify areas for further methods research and to provide guidance to researchers.

## Methods and materials

### Eligibility criteria *for* inclusion in the review

Included studies measured interobserver variability for an imaging test; were primary studies (no systematic review or methods paper); using real imaging test results (no artificial or modified images); with human patients; and were reported in English. There was no restriction on the clinical area of the interobserver variability studies.

### Search strategy

The Medline and Embase databases were searched for eligible studies published from January 2019 to January 2020 (see Supplementary Table 1 for search strategy). The search strategy was developed using medical subject headings and text words for interobserver variability and diagnostic imaging tests. The search strategy was used to identify studies with a variability term in the title. Conference abstracts were also included and summarised separately to full-text articles.

Supplementary Materials

PRISMA-DTA Checklist

### Article selection and data extraction

Screening for eligibility was completed independently for all studies in duplicate (by two different reviewers separately), any disagreements were discussed, and an additional reviewer was available for further discussion regarding eligibility.

Data extraction was completed for all studies by one reviewer. The first ten eligible studies were used to pilot the data extraction form and were included in the review. In addition, for a random sample of studies (10%), a second reviewer independently extracted data. The agreement between the two reviewers was good across data extraction items. Any disagreements were discussed and a consensus was reached. The data extraction form is reported in Supplementary Table 2.

### Risk of bias

The COSMIN risk of bias tool for assessing reliability and measurement error^8^ (see Supplementary Table 3-5) was developed for variability studies, including imaging tests. The tool was used in this review to assess the risk of bias for each study.Information on the imaging test, the research question, and the conduct of the study were used to check if the study design and method standards were met. Each standard was scored on a four-point scale (very good, adequate, doubtful and inadequate), with the lowest score giving the overall risk of bias for a study. The risk of bias tool was only used on full-text studies as not enough information was available in the conference abstracts.

### Data analysis

Basic summaries of extracted information were reported. Categorical variables were summarised using frequencies and percentages per category and for continuous variables, the mean and standard deviation or median and interquartile range (IQR) were reported. All analyses were completed in Stata version 16.1.

## Results

**Figure 1.**
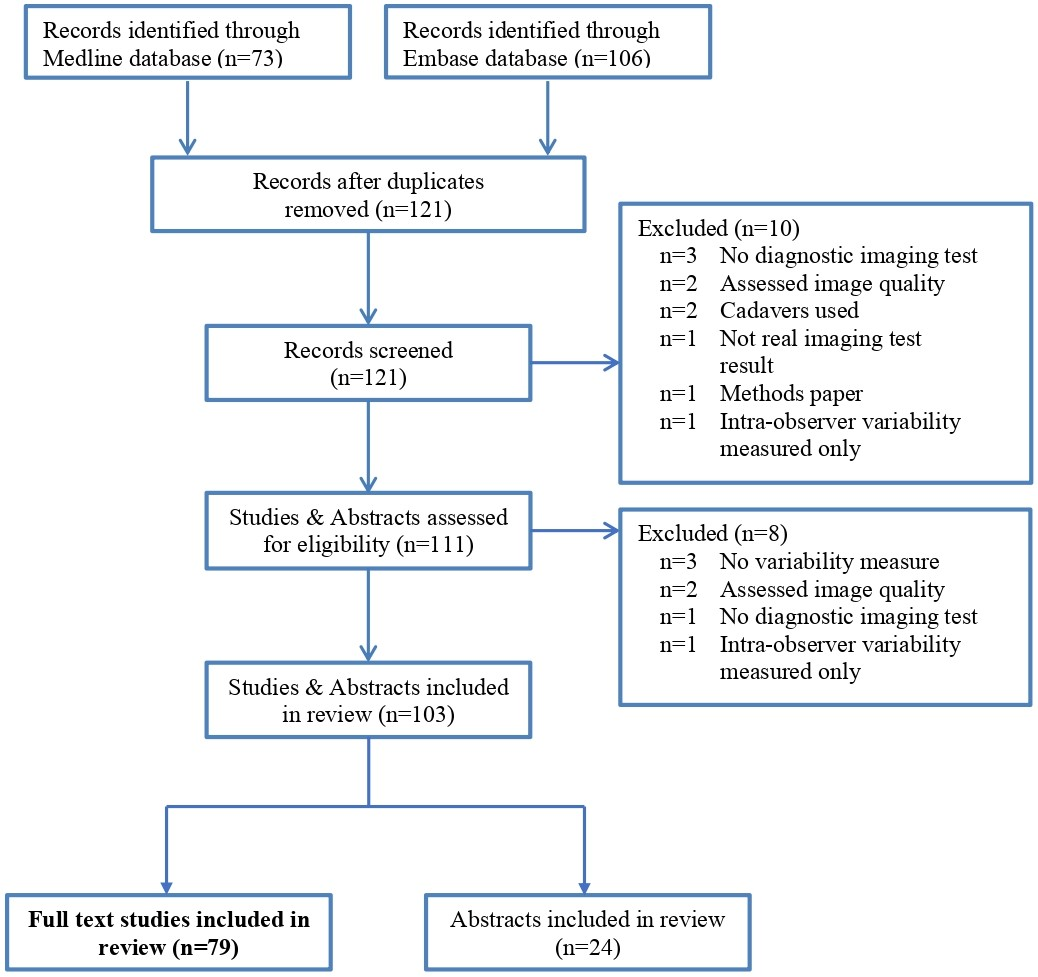
Flow diagram of systematic review

Seventy-nine full-text studies were included (Figure 1, see Supplementary Table 6 for author and titles). Studies were published in specialist radiology (*n* = 34, 43%) or clinical (*n* = 45, 57%) journals. Sixty-two studies (78%) focused primarily on interobserver variability [Table 1]. Fifty-one studies (65%) used retrospective data from hospital records, and 26 studies (33%) prospectively enrolled patients. In all studies, images were assessed prospectively, regardless of how the study participants were recruited. Cancer was the most common clinical area (*n* = 33, 42%) and MRI (*n* = 30, 38%) was the most common imaging test used. In addition, 24 conference abstracts were described in *Supplementary material 1*, characteristics reported were similar to the full-text studies.

**Table 1.** Main characteristics of the 79 studies

Characteristic	No. (%)
**Aim of study**	
Single aim	62 (78)
Multiple aims	17 (22)
**Patient data collection**	
Prospective	26 (33)
Retrospective	51 (65)
Mixture	1 (1)
Unknown	1 (1)
**Clinical areasa**	
Cancer	33 (42)
Muscle, joint and tendon problems	17 (22)
Liver conditions	5 (6)
Heart conditions	4 (5)
Eye conditions	3 (4)
**Imaging tests^+^**	
MRI	30 (38)
Ultrasound	17 (22)
CT	13 (17)
**Additional information on imaging test**	54 (68)
**Multiple imaging tests or versions of test**	23 (29)

a Clinical areas listed if two or more studies **^+^** Main imaging tests listed only

### Study design and sample size

In 75 (95%) of the 79 studies, all observers interpreted all images (sometimes known as a crossed design); in two studies, there was two health conditions evaluated so half of the observers interpreted images for each condition and in one study,^9^ only a subset of study observers interpret all images (sometimes known as a nested design). In 67 studies (85%), observers only repeated the interpretation of the same image for a patient (referred to as static images); in 11 studies (14%), observers repeated both the process of performing the imaging test and the interpretation of images (referred to as repeated imaging); one study did not provide this detail. Repeated imaging for interobserver variability studies is completed when there could be variability in the process of performing the test as well as the interpretation.

The number of patients varied greatly (median: 47, IQR 23 to 88), the smallest study had one patient^10^ and the largest study had 375 patients,^11^ see Table 2. Only 10 studies (13%) gave justification for their sample size; 7 (70%) studies gave a sample size calculation and 3 (30%) referred to the size of previous studies.^12–14^ The median number of observers was 4 (IQR 2 to 7) and ranged from 2 to 57.^9^ Twenty-one studies (27%) had two observers and 16 studies (20%) had three observers.Most studies had both a low number of observers and patients (*see Supplementary material 2*). For studies with more than 30 observers, these studies also had less than 50 patients. For studies with a high number of patients, there were a lower number of observers (almost all studies with over 50 patients had less than 10 observers).

**Table 2.** Patient and observer population characteristics of the 79 studies

Characteristic	No. (%)
	
**Patient population**	
**Sample size**	
Sample size – median (IQR^a^)	47 (23 to 88)
Sample size justification	12 (15)
**Recruiting centres**	
Single centre	51 (65)
Multiple centres	10 (13)
Unknown number of centres	18 (23)
**Population type**	
Condition only	55 (70)
Mixture	5 (6)
Healthy patient only	14 (18)
Unknown population type	5 (6)
**Reference standard for condition used**	39 (49)
**Inclusion criteria reported**	71 (90)
	
**Observer population**	
**Sample size** – median (IQRa)	4 (2 to 7)
**Recruiting centres**	
Single centre	26 (33)
Multiple centres	23 (29)
Unknown number of centres	30 (38)
**Experience**	
Experienced	20 (25)
Mixture	52 (66)
Inexperienced	2 (3)
Unknown	5 (6)
**Specific training given**	23 (29)

a IQR: Interquartile range

Details on recruitment methods for patients were scarce: 32 studies (41%) gave no details, 16 studies (20%) consecutively recruited or identified eligible patients, 13 studies (16%) selectively recruited patients, and 10 studies (13%) stated recruitment was reported in another study article. Fifty-five studies (70%) included only patients with the condition being investigated (diagnosis and staging), five studies (6%) included only healthy volunteers to obtain normal reference values or ranges for future studies and 14 studies (18%) included patients both with and without the condition being evaluated.

### Variability measures and their interpretation

Forty-nine studies (62%) evaluated continuous condition measurements, and 30 studies (38%) evaluated categorical condition measurements; however, over half of the included studies (*n* = 54, 68%) included multiple condition measurements, for example, length and area of tumour [Table 3]. Forty-one studies (52%) reported Intraclass correlation coefficients (ICC), of which 19 studies (46%) reported the type of ICC used. Thirty-one studies (39%) reported Kappa (κ) statistics, such as Cohen’s, Fleiss’s, Light’s and weighted κ [see Supplementary Table 7 for list]. Forty-four studies (56%) justified their interpretation of the variability estimate with a reference or clinical opinion, for example referring to the Landis and Koch interpretation of κ [15].

**Table 3.** Measurement evaluated and variability measures in the 79 studies

Characteristic	No. (%)
	
**Measurement evaluated**	
**Data type**	
Numeric	49 (62)
Categorical	30 (38)
**Multiple measurements evaluated**	54 (68)
	
**Variability measures**	
**Main type of variability measuresa**	
Intraclass correlation coefficients (ICC)	41 (52)
Kappa (κ) statistics	31 (39)
Limits of agreement	16 (20)
Percentage agreement	15 (19)
Coefficient of variation	11 (14)
Standard error of measurement	4 (5)
**Confidence intervals reported**	48 (61)
**Interpretation of variability measure justified**	35 (44)

a Studies can include multiple variability measures and all measures are not listed here

Thirty-eight studies (48%) had a positive conclusion, characterised as a recommendation for test use or an indication of low interobserver variability. For example, a study by Razek^15^ states that the imaging test is a “reliable and reproducible method for the diagnosis of central vein stenosis in haemodialysis patients”. Nineteen studies (24%) had a negative conclusion, stating the test should not be recommended for use or indicated a high interobserver variability. For example, a study by Apolle^16^ states that “Consensus guidelines are urgently needed”. Fourteen of the 19 studies (74%) with negative conclusions reported suggestions on how to improve the interobserver variability, such as further research, training or guidelines for taking or interpreting images. The other 22 studies (28%) did not clearly state whether or not they would recommend the imaging test.

The ICC and κ values varied largely across the different conclusions with no noticeable difference in the values leading to a positive, negative or mixed conclusion regarding recommending the test and/or test variability. The percentage agreement also varied across conclusions but was highest for positive conclusions followed by studies with mixed conclusions. For studies with positive conclusions, κ values ranged from −0.05 to 1.00, ICC values ranged from 0.14 to 0.99 and percentage agreement ranged from 75% to 100%.

### Assessment of COSMIN risk of bias tool

**Figure 2.**
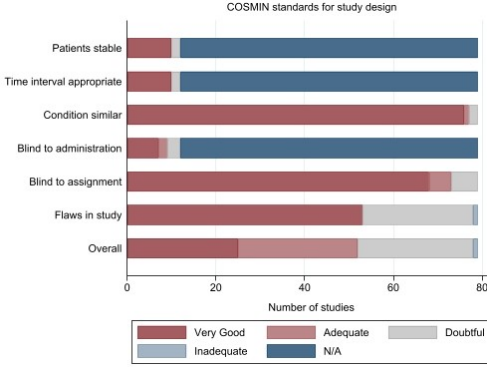
COSMIN Risk of Bias tool – Standards for study design. N/A – Standard is not applicable for study.

Of 79 studies, the overall ratings for risk of bias were ‘very good’ for 25 studies (32%), ‘adequate’ for 27 studies (34%), ‘doubtful’ for 26 studies (33%) and ‘inadequate’ for one study (1%) [Figure 2]. For studies that used static images (*n* = 67, 85%), standards on the stability of patients between measurements, on appropriate time intervals and observer blinding for taking the imaging test were rated as ‘not applicable’, according to the COSMIN manual. For the standard of assessing an image without knowledge of repeated results, most studies had a rating of ‘very good’ (*n* = 68, 86%), and the rest had ‘adequate’ (*n* = 5, 6%) or ‘doubtful’ (*n* = 6, 8%) rating. In most studies with a repeated generation of images (repeated imaging), measurements were performed on the same day. Most studies stated whether the interpretation of images was done without the knowledge of other test results but did not report if there was knowledge of performing the imaging test.

Of the 41 studies that reported ICCs, COSMIN ratings were ‘very good’(with the type of model reported) for 14 studies (34%) and were ‘adequate’for 27 studies (66%). Of the 11 studies that reported ordinal κ, ratings were ‘very good’ for four studies (36%) and were ‘adequate’ for seven studies (64%). Of the 31 studies that reported κ, ratings were ‘very good’ for all studies. Of the 23 studies that reported limits of agreement, coefficient of variation or standard error of measurement, ratings were ‘very good’ for 20 studies (87%) and ‘adequate’ for three studies (13%). Of the 15 studies that reported percentage agreement, ratings were ‘very good’ for all studies.

## Discussion

This review identified 79 studies and 24 conference abstracts using a search, covering one year from January 2019 to January 2020, across medical journals; a previous review included only 20 studies from four radiology journals from 2011 to 2012. We found sample sizes were often small without justification, and most studies reported ICC and κ values, which did not always align with study conclusions. Our review is one of the first to use the COSMIN risk of bias tool for assessing studies of imaging tests.^8^

### Study design and sample size

Static images are used in the majority of studies, so results only give information about the variability of the interpretation of the images, rather than the variability of the process of taking the imaging test (repeated imaging). Generally, imaging test results were retrospectively collected from hospital records and sent to observers to prospectively interpret the images. The advantages of using static images are costs and availability, the disadvantages are possible substandard images (as they may be older images from hospital records), selection bias (the use of retrospective images as opposed to prospectively collected images for repeated imaging)^17^and the inability to assess if there is variability the process of taking the imaging test. The disadvantages of using repeated imaging are that it can be expensive, expose patients to unnecessary radiation and can be difficult to perform (need to have patients and different observers available for multiple imaging tests).

Depending on the aim of the study, different study populations are appropriate. The most common study population is where all participants have the condition being investigated, and the measurement is condition-related (*n* = 55, 70%). The next most common study population is where all participants have symptoms suggestive of a condition (may or may not be diagnosed), participants without the condition are needed to see how often observers would agree on a diagnosis (*n* = 14, 18%). Some studies only had healthy participants, used to calculate reference values for a condition measurement or as a feasibility study before evaluating variability on participants with the condition (*n* = 5, 6%).

Study patient and observer populations may not be generalisable to clinical practice. This may be due to the study population chosen or the inability to verify generalisability due to poor reporting of patient and observer characteristics. The study population chosen and the variability within the study population can affect variability measures, making the selection of an appropriate study population (reflective of the population the test would be used in) critical.^6,7^ The number of patients included is generally small with no sample size justification and patients are recruited from a single centre (*n* = 51, 56%), which may not be generalisable. Most studies reported a mixture of experience for the study observers, but due to the low numbers of participating observers (often two or three), it is unlikely they are representative of observers in clinical practice. Allowing for a mixture of observers (different pairs of observers) interpreting images allows for a bigger sample size and a more representative sample. Having representative observers can mean having observers with a range of experience or experienced observers only, the observers just need to replicate those in clinical practice. It is preferable that patients and observers are from multiple centres to be representative and further research needs to be done on sample size calculations for interobserver variability studies.

### Variability measures and their interpretation

ICC and κ statistics were most frequently used, but specialist variability measures were used in some studies, such as overlap measures for contouring a tumour or organ.^18^In this review, interobserver variability was used as an umbrella term covering reliability and measurement error (which are two related but distinct measurement properties as defined in the GRRAS guidelines^6^ and the COSMIN risk of bias tool.)^8^

**Figure 3.**
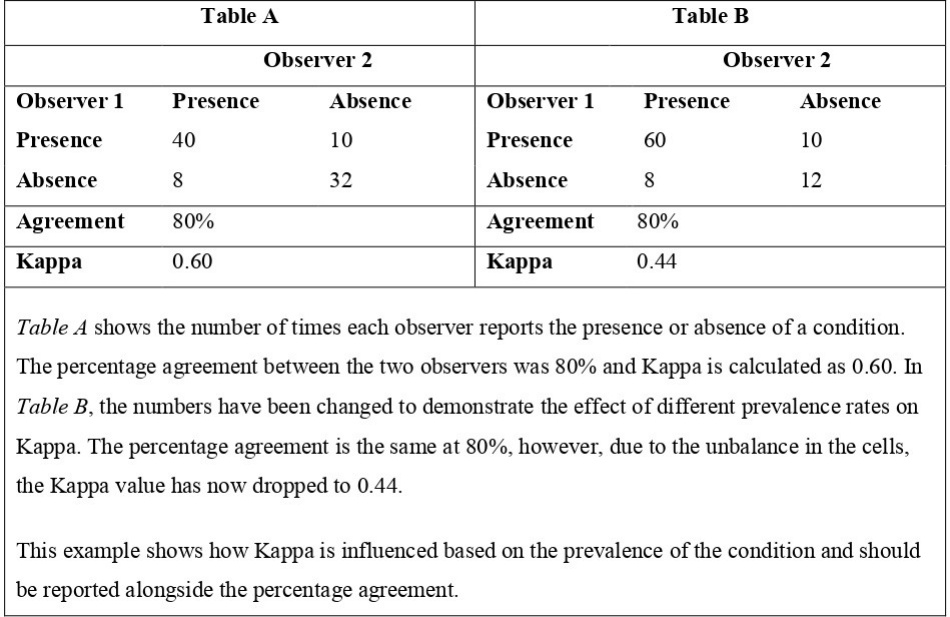
Example of the effect of marginal probabilities on κ statistics using a hypothetical example looking at the interobserver variability of an imaging test to detect the presence of a condition.

There are many methodological issues with κ statistics, regarding its assumptions, calculation and interpretation. These issues have been documented by various authors.^19–21^ The κ statistic (a reliability measure) is calculated based on marginal probabilities and influenced by the condition prevalence. In studies where the prevalence of the condition is very low or high, a high percentage agreement between observers can still lead to a very low κ [*see* Figure 3 *for example*], which can make the interpretation of results challenging [*see* Table 4]. There are some variability measures, adaptions of κ statistics created to account for the issue regarding prevalence, such as Gwet’s index,^22^ that are not widely used. Most studies interpret κ using the Landis and Koch interpretation, described by the authors as an arbitrary benchmark for the example they used in their paper.^23^ The cut-off points used split results into poor, slight, moderate, substantial and almost perfect, with no connection to clinical importance. Results from interobserver variability studies usually state κ values and their interpretations, without percentage agreement, which has an easier interpretation, or the numbers of interpretations for each category by observers for recalculation of variability estimates. Due to the issues regarding the effect of prevalence on κ statistics and the difficulty in its interpretation, we recommend that studies report a type of κ statistic (reliability measure), percentage agreement (measurement error) and the raw data in contingency tables if possible to ease the interpretation and allow for the calculation of different variability measures. The presentation of contingency tables is also recommended in diagnostic test accuracy studies for the calculation of different measures.^24^ In some studies, graphs were reported alongside variability estimates which can make results from interobserver variability studies easier to interpret (*e.g.,* Bland-Altman plots, scatterplots and bar charts).

**Table 4.** Example studies for interpretation of κ statistics

Issue	Study example	Description
**No issues**	Razek et al measuring interobserver variability of colour duplex ultrasound for detecting central vein stenosis in haemodialysis patients.	Percentage agreement between the two observers was 90% and κ was 0.84. Interpretation of the κ value would be almost perfect reliability which is similar to the interpretation of the percentage agreement reported. However, whether this is considered an acceptable level of agreement should always be based on clinical importance
**Minor issues**	Pierce et al measuring interobserver variability of MRI for detecting neurovascular bundle involvement in paediatric patients and young adults.	Percentage agreement between the two observers was 80% (95% CI: 72 to 88%) and κ was 0.60 (95% CI: 0.43 to 0.77) , which would be interpreted as moderate agreement. The 95% confidence intervals show that the estimates vary largely. The acceptable level of agreement should be based on clinical importance and could be made difficult by the difference in the variability estimates.
**Major issues**	Castro et al measuring interobserver variability of ultrasound for measurements of patellar and quadriceps tendons in critically ill patients.	For proximal quadricep measurements, percentage agreement was 75% and κ was 0.31 (interpreted as slight agreement). For distal patellar measurements, percentage agreement was 90% and κ was −0.05 (interpreted as poor agreement). The percentage agreement values suggest agreement was quite high, however, the arbitrary cut-off points usually used for κ suggest slight and poor agreement. Like previous, examples an acceptable level of agreement should be based on clinical importance but the large difference between the values make interpreting these results very difficult.

The discussion and conclusions of studies do not always align with the results given in interobserver variability studies. Variability estimates vary largely, regardless of the type of conclusion stated (positive, mixed or negative). A minimum requirement appropriate to the clinical situation should be considered for variability estimates when deciding on the use of an imaging test and an explanation of why this is considered sufficient so that the results and conclusion align with each other. Primary outcomes should also be decided in advance, this can help with aligning conclusions to results as many studies measure interobserver variability for multiple condition measurements.

### Risk of bias

The COSMIN risk of bias tool standards relates to the imaging test but not to whether the included patients represent the population of interest or how similar the variability estimate is to clinical practice. Overall, the majority of studies had a ‘very good’ or ‘adequate’ rating (66%). For study design standards, some were not applicable due to the use of static images, but these did not influence the overall rating of a study. For the standard on flaws, a list was created [see *Supplementary material 3*].

Only standards on the preferred statistical methods for commonly used variability measures (*e.g.,* ICC, κ for reliability and SEM or percentage agreement for the measurement error) are given in the COSMIN tool. We found additional measures (*i.e.,* for tumour contouring) for which no standards were provided. Further research is needed to recommend when different variability measures should be used.

A check of the most commonly included journals in the review showed that while there is a recommendation to use guidelines in some journals, they are not required. The standard of paper across all of the journals varied according to the risk of bias tool.

### Strengths and limitations

Compared to a previous review in this area, this review includes a substantive sample of studies and conference abstracts across medical journals giving a more representative sample^7^ and the methods for this review are robust (a detailed protocol has been published,^25^ screening eligibility was performed in duplicate independently and a pilot was completed to minimise the risk of errors).

The search strategy required a variability term in the title as it was expected these studies would befocussed on interobserver variability and therefore likely to report more information on the interobserver variability study design and methods as found in a previous review.^7^However, there could be studies focussed on interobserver variability that were missed which did not have a variability term in the title. This was decided so sufficient information on study design and methods was available.This review included only one recent year of publications and only reported in English due to resource constraints.

### Recommendations

A list of recommendations for designing and conducting interobserver variability studies based on the results of this review and previous guidelines (GRRAS and COSMIN) are listed [Table 5].

**Table 5.** Recommendations for interobserver variability studies

		Recommendation
Study design	Static image *vs* repeated imaging	For imaging tests with possible variability in the process of generating the image, repeating the imaging test should be considered for an accurate variability estimate.
	Observers	A mixture of observers (different pairs of observers) interpreting images for different patients allows for a bigger sample size and a more representative sample.
	Sample size	Sample size calculations or justifications for the number of patients and observers should be given
	Representativeness	Patients and observers representative of clinical practice allows for a more realistic estimate of variability, preferably from multiple centres
Methods	Outcomes	Primary outcomes (measurements) should be established a priori.
	Variability measures	For categorical measures, percentage agreement should be reported alongside κ statistics or adaptions of κ statistics.
Results	Results	The raw data such as contingency tables should be given to allow the reader to calculate different variability estimates, where applicable
	Conclusions	Decisions on whether a diagnostic imaging test is recommended should be based on clinical importance and not applying arbitrary cut-off points to results.
Guidelines	Reporting and risk of bias	GRRAS guidelines (1) can be used for reporting interobserver variability studies and the COSMIN risk of bias tool (2) can be used to assess the quality of studies.

## Conclusions

In conclusion, interobserver variability studies have diverse study designs and methods. We identified issues with study design and sample size, choice of variability measures and their interpretation, this review has identified the need for further research and guidance for researchers. Some recommendations were proposed in the interim.

## References

[1] Itri JN, Tappouni RR, McEachern RO, Pesch AJ, Patel SH . Fundamentals of diagnostic error in imaging. RadioGraphics 2018; 38: 1845–65. doi: 10.1148/rg.2018180021 30303801

[2] Bruno MA, Walker EA, Abujudeh HH . Understanding and confronting our mistakes: the epidemiology of error in Radiology and strategies for error reduction. Radiographics 2015; 35: 1668–76. doi: 10.1148/rg.2015150023 26466178

[3] Sardanelli F, Di Leo G . *Biostatistics for Radiologists* . Milano: Springer Science & Business Media; 2009. doi: 10.1007/978-88-470-1133-5

[4] Fraser CG, Harris EK . Generation and application of data on biological variation in clinical chemistry. Crit Rev Clin Lab Sci 1989; 27: 409–37. doi: 10.3109/10408368909106595 2679660

[5] de Vet HCW, Terwee CB, Knol DL, Bouter LM . When to use agreement versus reliability measures. Journal of Clinical Epidemiology 2006; 59: 1033–39. doi: 10.1016/j.jclinepi.2005.10.015 16980142

[6] Kottner J, Audige L, Brorson S, Donner A, Gajewski BJ, Hróbjartsson A, et al . Guidelines for reporting Reliability and agreement studies (GRRAS) were proposed. International Journal of Nursing Studies 2011; 48: 661–71. doi: 10.1016/j.ijnurstu.2011.01.016 21514934

[7] Farzin B, Gentric J-C, Pham M, Tremblay-Paquet S, Brosseau L, Roy C, et al . Agreement studies in Radiology research. Diagn Interv Imaging 2017; 98: 227–33. doi: 10.1016/j.diii.2016.05.014 27473190

[8] Mokkink LB, Boers M, van der Vleuten CPM, Bouter LM, Alonso J, Patrick DL, et al . COSMIN risk of bias tool to assess the quality of studies on reliability or measurement error of outcome measurement instruments: a Delphi study. BMC Med Res Methodol 2020; 20: 293: 293. doi: 10.1186/s12874-020-01179-5 33267819 PMC7712525

[9] El-Tawil S, Mair G, Huang X, Sakka E, Palmer J, Ford I, et al . Observer agreement on computed tomography perfusion imaging in acute ischemic stroke. Stroke 2019; 50: 3108–14. doi: 10.1161/STROKEAHA.119.026238 31928512 PMC6824508

[10] Trignani M, Argenone A, Di Biase S, Musio D, Merlotti A, Ursino S, et al . Inter-observer variability of clinical target volume delineation in definitive radiotherapy of neck lymph node metastases from unknown primary. A cooperative study of the Italian Association of radiotherapy and clinical oncology (AIRO). Radiol Med 2019; 124: 682–92. doi: 10.1007/s11547-019-01006-y 30852793

[11] Zabala-Travers S, Gallas BD, Busoni S, Williams MC, Noferini L, Fedeli L, et al . Display colour scale effects on diagnostic performance and reader agreement in cardiac CT and prostate apparent diffusion coefficient assessment. Clin Radiol 2019; 74: 79. doi: 10.1016/j.crad.2018.08.016 30336942

[12] Mosher TJ, Liu Y, Torok CM . Functional cartilage MRI T2 mapping: evaluating the effect of age and training on knee cartilage response to running. Osteoarthritis and Cartilage 2010; 18: 358–64. doi: 10.1016/j.joca.2009.11.011 19948266 PMC2826588

[13] McAlinden C, Khadka J, Pesudovs K . Precision (Repeatability and reproducibility) studies and sample-size calculation. Journal of Cataract and Refractive Surgery 2015; 41: 2598–2604. doi: 10.1016/j.jcrs.2015.06.029 26796439

[14] Shank CF, Wiater BP, Pace JL, Jinguji TM, Schmale GA, Bittner RCL, et al . The lateral Capitellohumeral angle in normal children: mean, variation, and reliability in comparison to Baumann’s angle. J Pediatr Orthop 2011; 31: 266–71. doi: 10.1097/BPO.0b013e31821009af 21415685

[15] Razek AA, Khalek AM, Elwakeel H, Ebrahim MA . Inter-observer agreement of color duplex ultrasound of central vein stenosis in Hemodialysis patients. Phlebology 2019; 34: 636–42. doi: 10.1177/0268355519837048 30871440

[16] Apolle R, Appold S, Bijl HP, Blanchard P, Bussink J, Faivre-Finn C, et al . Inter-observer variability in target delineation increases during adaptive treatment of head-and-neck and lung cancer. Acta Oncologica 2019; 58: 1378–85. doi: 10.1080/0284186X.2019.1629017 31271079

[17] Talari K, Goyal M . Retrospective studies–utility and Caveats. Journal of the Royal College of Physicians of Edinburgh 2020; 50: 398–402. doi: 10.4997/jrcpe.2020.409 33469615

[18] Fotina I, Lütgendorf-Caucig C, Stock M, Pötter R, Georg D . Critical discussion of evaluation parameters for inter-observer variability in target definition for radiation therapy. Strahlenther Onkol 2012; 188: 160–67. doi: 10.1007/s00066-011-0027-6 22281878

[19] Flight L, Julious SA . The disagreeable behaviour of the Kappa statistic. Pharmaceut Statist 2015; 14: 74–78. doi: 10.1002/pst.1659 25470361

[20] Dettori JR, Norvell DC . Kappa and beyond: is there agreement Global Spine Journal 2020; 10: 499–501. doi: 10.1177/2192568220911648 32435572 PMC7222679

[21] Feinstein AR, Cicchetti DV . High agreement but low Kappa: I. the problems of two Paradoxes. J Clin Epidemiol 1990; 43: 543–49. doi: 10.1016/0895-4356(90)90158-l 2348207

[22] Wongpakaran N, Wongpakaran T, Wedding D, Gwet KL . A comparison of Cohen’s Kappa and Gwet’s Ac1 when calculating inter-Rater reliability coefficients: a study conducted with personality disorder samples. BMC Med Res Methodol 2013; 13(): 61. doi: 10.1186/1471-2288-13-61 23627889 PMC3643869

[23] Landis JR, Koch GG . The measurement of observer agreement for categorical data. Biometrics 1977; 33: 159. doi: 10.2307/2529310 843571

[24] Cohen JF, Korevaar DA, Altman DG, Bruns DE, Gatsonis CA, Hooft L, et al . STARD 2015 guidelines for reporting diagnostic accuracy studies: explanation and elaboration. BMJ Open 2016; 6: e012799. doi: 10.1136/bmjopen-2016-012799 PMC5128957 28137831

[25] Quinn L, Sitch A, Deeks J, et al . Interobserver variability in diagnostic imaging: A methodological systematic review protocol. OSF 2020. 10.1259/bjr.20220972 PMC10392644 37399082

