# Supplementary material for: Interobserver variability studies in diagnostic imaging: a methodological systematic review
Source: Br J Radiol. 2023 Jun 29;96(1148):20220972. doi: 10.1259/bjr.20220972 (PMC10392644; doi:10.1259/bjr.20220972)
Supplement: Supplementary Materials [file bjr.20220972.suppl-01.docx]

**Supplementary material**

**Supplementary material 1** – Summary of conference abstract characteristics

Twenty-four conference abstracts of studies were identified. Due to the limited information available, only key information was extracted and summarised. Twenty-two conference abstracts (92%) primarily focussed on interobserver variability. Seven studies (29%) used retrospective data from hospital records and nine studies (33%) prospectively enrolled patients. Information of data collection or population sources was not available for the other abstracts. Cancer was the most common clinical area and MRI was the most common imaging test used.

*Study design and sample size*

In 21 conference abstracts (88%) all observers interpreted all images. The median number of patients was 25 (IQR 9 to 67), no justification of sample size was given. The median number of observers was three (IQR 2 to 6). Of the abstracts with information on observer experience, seven studies (29%) had experienced observers only and four studies (17%) had observers with a mixture of experience. Most abstracts included only patients with condition being investigated, one abstract included patients with and without the condition.

*Variability measures and their interpretation*

Fifteen abstracts (63%) evaluated continuous measurements, over half of the abstracts (n=13, 54%) included multiple measurements. ICC and Kappa statistics were the most common variable measures reported. Other variability measures reported included percentage agreement, Krippendorff alpha, coefficient of variation and dice similarity coefficient. The majority of abstracts (17, 71%) had positive conclusions characterised as a recommendation for the use of the test. Most abstracts reported no limitations (22, 92%).

## Supplementary material 2 – Scatterplot of number of patients and observers per study


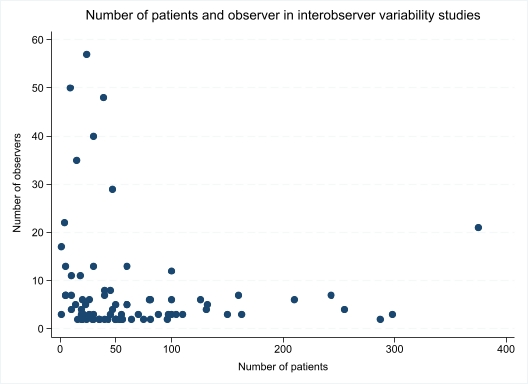


**Supplementary material 3–** List of flaws for design standard

When using the COSMIN risk of bias tool, these were the flaws that would give an adequate/doubtful/inadequate rating:

- a study that has less than 10 patients or only two observers may not be representative of a wider population to get a proper estimate of interobserver variability
- having two observers for a study where observers repeated the whole imaging process was not considered a flaw as this could be due to resource constraints and patient availability.
- studies where multiple observers interpret a static image and one of those observers also administers the test, mean that the observer who administers the test would have extra information not available to other observers and so potentially bias the results.

## Supplementary table 1 - Medline search strategy

| **#** | **Searches** |
| --- | --- |
| 1 | inter AND (observer OR examiner OR reader OR rater) [Title] |
| 2 | reliab* AND (observer OR examiner OR reader OR rater) [Title] |
| 3 | variation OR variability AND (observer OR examiner OR reader OR rater) [Title] |
| 4 | agreement AND (observer OR examiner OR reader OR rater) [Title] |
| 5 | 1 OR 2 OR 3 OR 4 |
| 6 | diagnostic imaging [MeSH Terms] |
| 7 | imaging [All fields] |
| 8 | 6 OR 7 |
| 9 | 5 AND 8 |
| 10 | Limit 9 to publications from Jan 2019 to Jan 2020 |
| 11 | Limit 10 to human participants AND English only |

## Supplementary table 2 – Data extraction form

| **Characteristics of studies included in review** | | | **Description** |
| --- | --- | --- | --- |
| A: Main study characteristics | | | |
| A1 | | Main outcome  [yes, if no state other outcomes] |  |
| A2 | | Type of data collection  [prospective, retrospective, other, unknown] |  |
| A3 | | Terminology used**  [agreement, variability, reliability] |  |
| A4 | | Clinical topic  [related to measurement being taken] |  |
| B: Imaging test | | | |
| B1 | | Type of imaging test**  [MRI, US, CT, etc.] |  |
| B2 | | Further details of imaging test and related equipment  [3-D ultrasound, ultrasound gel, etc.] |  |
| B3 | | Version of imaging test reported  [yes, no]** |  |
| B4 | | Is this how test is typically used in practice  [yes, no, unknown] |  |
| B5 | | More than one imaging test  [yes, no] |  |
| B5a | | If yes, number of imaging tests |  |
| B5b | | If yes, name of other imaging tests |  |
| C: Sample population | | | |
| C1 | Number of patients | |  |
| C2 | Reference standard or true diagnosis available  [yes/no and free text explanation] | |  |
| C3 | Patient population  [condition only, mixture, healthy, unknown/not reported]** | |  |
| C4 | Single/Multicentre study for patients  [unknown] | |  |
| C5 | Sample source reported  [hospital, area, country] | |  |
| C6 | Patients imaging representative of those to whom the results intended to be applied  [yes, no, unknown] | |  |
| C7 | Sample selection method  [consecutive, selective, from existing study or trial, incentivised, other, unknown] | |  |
| C8 | Sample size justification  [yes, no, unknown] | |  |
| C8a | If justification, type  [power calculation, previous study, reference] | |  |
| C9 | Inclusion criteria reported  [yes, no] | |  |
| C10 | Preparatory action taken by patients | |  |
| C10a | If preparatory action, adherence to instructions | |  |
| D: Observers | | | |
| D1 | Number of observers | |  |
| D2 | Experience of observers  [experienced, inexperienced, mixture, unknown]* | |  |
| D3 | Single/Multicentre study for observers  [unknown] | |  |
| D4 | Specific training for observers during study  [yes, no, unknown]* | |  |
| D5 | Observers representative of those in practice  [yes, no, unknown] | |  |
| D6 | Did all observers interpret results for all patients? [yes, no, unknown] | |  |
| D6a | If no, description of study design  [number of observers per patient] | |  |
| D7 | Preparatory action taken by observer reported* | |  |
| D7a | Preparation of device/equipment* | |  |
| D7b | Preparations or requirements for environmental conditions* | |  |
| D7c | Provide general and preparatory instructions to the patient* | |  |
| D7d | Preparatory actions on the patient* | |  |
| D7e | Performance of familiarization sessions, if required* | |  |
| D8 | Raw data collection by observer* | |  |
| E: Possible bias in study design | | | |
| E1 | If true diagnosis available, observers independent and blind to prevalence of condition in study [yes, no, unknown] | |  |
| E2 | If true diagnosis available, observers independent and blind to final diagnosis [yes, no, unknown] | |  |
| E3 | Observers independent and blind to clinical information [yes, no, unknown] | |  |
| E4 | Observers blind to additional cues that were not part of test [yes, no, unknown] | |  |
| E5 | Observers blind to previous findings of test under evaluation [yes, no, unknown] | |  |
| E6 | Observers independent and blind to other observers [yes, no, unknown] ^+^ | |  |
| E7 | Order of examination varied [yes, no, unknown] | |  |
| E8 | Components of imaging interpretation repeated – Is the test repeated or images are stored [unknown]** | |  |
| E8a | If images stored, information on storage of data* | |  |
| E8b | If images redone, was the stability of measurement taking into account when determining time interval between examinations^+^ | |  |
| E8c | If images redone, time interval between examinations^+^ | |  |
| F: Measurement evaluated | | | |
| F1 | Unit of analysis for measurement  [patient, organ, lesion, multiple] | |  |
| F2 | Description of unit of analysis** | |  |
| F3 | Categorisation thresholds for unit of analysis* | |  |
| F4 | Data type of measurement being evaluated  [numeric, categorical] | |  |
| F4a | Information about measurement being evaluated* | |  |
| F5 | Multiple measurements being evaluated  [yes, no] | |  |
| F5a | If yes, number of measurements being evaluated [2, 3, ≥4, unknown] | |  |
| F5c | If yes, different data types of measurements | |  |
| G: Variability measure | | | |
| G1 | Type of variability measure used^+^ | |  |
| G1a | ICC used  [State analysis model if reported] | |  |
| G1b | Kappa used  [If yes, state type] | |  |
| G1c | Percentage agreement | |  |
| G1d | If other, state type of variability measure | |  |
| G2 | Analysis in study not related to variability measure [yes, no] | |  |
| G3 | Confidence intervals reported for variability estimates | |  |
| G4 | Is variability measure used appropriate?  [based on data type] | |  |
| G5 | Justification of interpretation of variability measure used [if yes, reference used?] | |  |
| G6 | Any subgroup analysis completed  [yes, no, unknown] | |  |
| G6a | If yes, was analysis justified  [free text] | |  |
| G7 | Characteristics of disagreement reported  [yes, no, unknown] | |  |
| G7a | If yes, methods used | |  |
| G8 | Intra-observer variability measured | |  |
| G8a | If intra-observer variability measured,  type of variability measure | |  |
| G8b | If intra-observer variability measured,  how many observers | |  |
| G8c | If intra-observer variability measured,  time between the measurements being taken | |  |
| H: Result presentation | | | |
| H1 | Patient characteristics reported  [age and sex are minimum] | |  |
| H2 | Observer characteristic reported  [years of experience, speciality, frequency of imaging reads, etc.] | |  |
| H3 | Raw data presented by observer or patient  [free text] | |  |
| H3 | Graphical presentation of results  [yes, no, unknown] | |  |
| H3a | If yes, description | |  |
| I: Discussion | | | |
| I1 | Interobserver variability mentioned in discussion | |  |
| I2 | Limitations stated | |  |
| I2a | If limitations, descriptions | |  |
| I3 | Overall conclusion | |  |
| I4 | Suggestions of improvement if variability is high [N/A, training, removal of test, other] | |  |

* Refers to **components of** **measurement instrument**

** Refers to **research question**

*** Refers to **design standards/methods standards**

## Supplementary table 3 – COSMIN risk of bias tool - Standards about design requirements for studies on reliability or measurement error

| **Design requirements** | | **very good** | **adequate** | **doubtful** | **inadequate** | **NA** |
| --- | --- | --- | --- | --- | --- | --- |
| 1 | Were patients stable in the time between the administration of the repeated measurements on the construct to be measured? | Evidence provided that patients were stable | Assumable that patients were stable | Unclear if patients were stable | Patients were NOT stable | Na |
| 2 | Was the time interval between the measurements appropriate? | Time interval appropriate |  | Doubtful whether time interval was appropriate or time interval was not stated | Time interval NOT appropriate | Na |
| 3 | Were the measurement conditions similar for the measurements – except for the condition being evaluated as a source of variation? | Measurement conditions were similar (evidence provided) | Assumable that measurement conditions were similar | Unclear if measurement conditions were similar | Measurement conditions were NOT similar | Na |
| 4 | Did the professional(s) administer the measurement without knowledge of scores of other repeated measurement(s) in the same patients? | Measurements administered without knowledge of other scores (evidence provided) | Assumable that measurements were administered without knowledge of other scores | Unclear if measurements were administered without knowledge of other scores | Measurements administered with knowledge of other scores |  |
| 5 | Did the professional(s) assign scores or determine values without knowledge of the scores of other repeated measurement(s) in the same patients? | Scores assigned or values determined without knowledge of other scores (evidence provided) | Assumable that scores were assigned or values were determined without knowledge of other scores | Unclear if scores were assigned or values were determined without knowledge of other scores | Scores were NOT assigned or values were NOT determined without knowledge of other scores |  |
| 6 | Were there any other important flaws in the design or statistical  methods of the study? | No other important methodological flaws |  | Other minor methodological flaws | Other important methodological flaws |  |

## Supplementary table 4 – COSMIN risk of bias tool - Standards for preferred statistical methods for reliability

| *Statistical methods* | | **very good** | **adequate** | | **doubtful** | | | **inadequate** |
| --- | --- | --- | --- | --- | --- | --- | --- | --- |
| 7 | For continuous scores: was an Intraclass Correlation Coefficient (ICC) or Generalizability (G) Coefficient calculated? | ICC or G Coefficient calculated; the model or formula was described, and matches the reviewer constructed research question and the data | ICC or G Coefficient calculated but model or formula was not described or does not optimally match the reviewer constructed research question  OR  Pearson or Spearman correlation coefficient calculated WITH evidence provided that no systematic difference between measurements has occurred | | Pearson or Spearman correlation coefficient calculated WITHOUT evidence provided that no systematic difference between measurements has occurred  OR WITH evidence provided that systematic difference between measurements has occurred | | |  |
| 8 | For ordinal scores: was a (weighted) kappa calculated? | Kappa calculated; the weighting scheme was described, and matches the reviewer constructed research question and the data | Kappa calculated, but weighting scheme not described or does not optimally match the reviewer constructed research question | |  | |  | |
| 9 | For dichotomous/nominal scores: was Kappa calculated for each category against the other categories combined? | Kappa calculated for each category against the other categories combined |  |  | |  | | |

**Supplementary table 5 –** COSMIN risk of bias tool - Standards for preferred statistical method of measurement error

| *Statistical methods* | | **very good** | **adequate** | **doubtful** | **inadequate** |
| --- | --- | --- | --- | --- | --- |
| 7 | For continuous scores: was the Standard Error of Measurement (SEM), Smallest Detectable Change (SDC), Limits of Agreement (LoA) or Coefficient of Variation (CV) calculated? | SEM, SDC, LoA or CV calculated; the model or formula for the SEM/SDC is described; it matches the reviewer constructed research question and the data | SEM, SDC, LoA or CV calculated, but the model or formula is not described or does not optimally match the reviewer constructed research question* and evidence provided that no systematic difference has occurred | SEM_consistency_ SDC_consistency_ or LoA or CV calculated, without knowledge about systematic difference or with evidence provided that systematic difference has occurred | SEM calculated based on Cronbach’s alpha, or using SD from another population |
| 8 | For dichotomous/nominal/ordinal scores: Was the percentage specific (e.g. positive and negative) agreement calculated? | % specific agreement calculated | % agreement calculated |  |  |

## Supplementary table 6 – Articles and conference abstracts included

| **First Author & year** | **Article title** |
| --- | --- |
| Anz 2020 | 3-T MRI mapping is a valid in vivo method of quantitatively evaluating the anterior cruciate ligament: rater reliability and comparison across age |
| Apolle 2019 | Inter-observer variability in target delineation increases during adaptive treatment of head-and-neck and lung cancer |
| Barchetti 2019 | Multiparametric MRI of the bladder: inter-observer agreement and accuracy with the Vesical Imaging-Reporting and Data System (VI-RADS) at a single reference center |
| Bartel 2019 | Inter-observer variation of hippocampus delineation in hippocampal avoidance prophylactic cranial irradiation |
| Basson 2019 | Chest Magnetic Resonance Imaging Decreases Inter-observer Variability of Gross Target Volume for Lung Tumors |
| Beals 2019 | Inter-rater reliability of TT-TG distance is good and does not vary based on preselected versus independent slice selection on MRI |
| Becker 2019 | Variability of manual segmentation of the prostate in axial T2-weighted MRI: A multi-reader study |
| Beer 2019 | Inter- and intra-reader agreement for gadoxetic acid-enhanced MRI parameter readings in patients with chronic liver diseases |
| Bignotti 2019 | Background parenchymal enhancement assessment: Inter- and intra-rater reliability across breast MRI sequences European Journal of Radiology |
| Brage 2019 | Ultrasonic strain elastography for detecting abnormalities in the supraspinatus tendon: An intra-and inter-rater reliability study |
| Bruin 2019 | Inter- and intra-observer variability in fetal ductus venosus blood flow measurements in high-risk fetuses at 26-32 weeks |
| Castro 2019 | Intra- And inter-rater reproducibility of ultrasound imaging of patellar and quadriceps tendons in critically ill patients |
| Chelu 2019 | Evaluation of atrial septal defects with 4D flow MRI-multilevel and inter-reader reproducibility for quantification of shunt severity |
| Chlebus 2019 | Reducing inter-observer variability and interaction time of MR liver volumetry by combining automatic CNN-based liver segmentation and manual corrections |
| Cocco 2019 | Inter-observer reliability of alternative diagnostic methods for proximal humerus fractures: A comparison between attending surgeons and orthopedic residents in training Patient |
| Corrao 2019 | Intra- and inter-observer variability in breast tumour bed contouring and the controversial role of surgical clips |
| Cummins 2019 | Establishing the inter-rater reliability of spinal cord damage manual measurement using magnetic resonance imaging |
| Daniel 2019 | Intra- and inter-observer variability in dependence of T1-time correction for common dynamic contrast enhanced MRI parameters in prostate cancer patients |
| Davaris 2019 | Flexible transnasal endoscopy with white light or narrow band imaging for the diagnosis of laryngeal malignancy: diagnostic value, observer variability and influence of previous laryngeal surgery |
| Dijkstra 2019 | Inter-observer reproducibility of quantitative dynamic susceptibility contrast and diffusion MRI parameters in histogram analysis of gliomas |
| Dondi 2020 | Inter-reader variability of SPECT MPI readings in low- and middle-income countries: Results from the IAEA-MPI Audit Project (I-MAP) |
| Dujardin 2019 | Perfusion Quantification of Liver Metastases of Colorectal Cancer Treated with Anti-angiogenic-Based Therapy: Assessment of Intra- and Inter-observer Reproducibility of Parameters in Three Regions of Interest Outlining Lesions |
| El-Tawil 2019 | Observer Agreement on Computed Tomography Perfusion Imaging in Acute Ischemic Stroke |
| Erkus 2019 | Intra- and inter-observer reliability of Laredo classification system in Legg-Calve-Perthes Disease |
| Federico 2019 | Prospective intra/inter-observer evaluation of pre-brachytherapy cervical cancer tumor width measured in trus and MR imaging International |
| Filippo 2019 | Inter-rater and intra-rater reliability of ultrasound imaging for measuring quadriceps muscle and non-contractile tissue thickness of the anterior thigh |
| Gaibazzi 2019 | Standard echocardiography versus very-low mechanical index contrast-imaging: left ventricle volumes and ejection fraction multi-reader variability and reference values in a subgroup with no risk factors or cardiac disease |
| Gansera 2019 | Utility of conventional aortic root shot angiography for SAPIEN 3 prosthesis sizing in TAVI: Feasibility and inter-reader variability |
| Garza-Leon 2020 | Comparison of meibomian gland loss area measurements between two computer programs and intra-inter-observer agreement |
| Growcott 2020 | Inter-Observer Variability in Target Volume Delineations of Benign and Metastatic Brain Tumours for Stereotactic Radiosurgery: Results of a National Quality Assurance Programme |
| Hague 2019 | Use of a novel atlas for muscles of mastication to reduce inter observer variability in head and neck radiotherapy contouring |
| Henrichon 2020 | Dynamic MRI of the wrist in less than 20 seconds: normal midcarpal motion and reader reliability |
| Hong 2019 | Reader agreement and accuracy of ultrasound features for hepatic steatosis |
| Hooker 2019 | Inter-reader agreement of magnetic resonance imaging proton density fat fraction and its longitudinal change in a clinical trial of adults with nonalcoholic steatohepatitis |
| Itani 2019 | Inter-observer Variability in the American College of Radiology Thyroid Imaging Reporting and Data System: In-Depth Analysis and Areas for Improvement |
| Joskowicz 2019 | Inter-observer variability of manual contour delineation of structures in CT |
| Kallis 2019 | Impact of inter- and intra-observer variabilities of catheter reconstruction on multi-catheter interstitial brachytherapy of breast cancer patients Radiotherapy & Oncology |
| Karakus 2019 | Is it difficult to obtain inter-observer agreement in the measurement of the beta angle in ultrasound evaluation of the paediatric hip? |
| Kim 2019 | Interpretive performance and inter-observer agreement on digital mammography test sets |
| Koh 2019 | Assessing sizes of breast cancers that show non-mass enhancement on MRI based on inter-observer variability and comparison with pathology size |
| Kohestani 2019 | Performance and inter-observer variability of prostate MRI (PI-RADS version 2) outside high-volume centres |
| Koo 2019 | Inter-rater reliability between experienced and inexperienced otolaryngologists using Koo's drug-induced sleep endoscopy classification system |
| Krabbe 2019 | Canada-Denmark MRI scoring system of the spine in patients with axial spondyloarthritis: Updated definitions, scoring rules and inter-reader reliability in a multiple reader setting |
| Larghi 2019 | Concordance, intra- and inter-observer agreements between light microscopy and whole slide imaging for samples acquired by EUS in pancreatic solid lesions Digestive and Liver Disease |
| Lea 2019 | Intra and inter-rater agreement of inflammatory choroidal neovascular membrane measurements using optical coherence tomography angiography |
| Lee 2019 | Investigating the intra- and inter-rater reliability of a panel of subjective and objective burn scar measurement tools |
| Mandell 2019 | A simplified classification of proximal femoral fractures improves accuracy, confidence, and inter-reader agreement of hip fracture classification by radiology residents |
| Mann 2020 | Tibial bone stress injury: diagnostic performance and inter-reader agreement of an abbreviated 5-min magnetic resonance protocol |
| Mossa-Basha 2019 | Inter-rater and scan-rescan reproducibility of the detection of intracranial atherosclerosis on contrast-enhanced 3D vessel wall MRI |
| Mouawad 2019 | Reducing the dose of gadolinium-based contrast agents for DCE-MRI guided SBRT: The effects on inter and intra observer variability for preoperative target volume delineation in early stage breast cancer patients |
| Musikachart 2019 | Intra-Observer and Inter-Observer Reliability of Shaft Condylar Angle and Lateral Capitellohumeral Angle: Evaluation Based on Reliability in Different Ages and Levels of Experience Orthopaedic Audio-Synopsis Continuing Medical Education |
| Nelson 2019 | Interobserver and Intra-Observer Reliability of the Urinary Tract Dilation Classification System in Neonates: A Multicenter Study |
| Nicholls 2020 | Inter- and intra-reader reproducibility of shear wave elastography measurements for musculoskeletal soft tissue masses |
| Oh 2019 | Inter-rater agreement among multiple examiners for the assessment of plaque scores between quantitative light-induced fluorescence-digital and two-tone disclosing solution-stained digital images |
| Papavero 2019 | Redundant nerve roots in lumbar spinal stenosis: inter- and intra-rater reliability of an MRI-based classification |
| Pierce 2019 | Inter-rater Variability in the Interpretation of Pre and Post Contrast MRI for Pre-Surgical Evaluation of Osteosarcoma in Long Bones in Pediatric Patients and Young Adults |
| Pirri 2019 | Inter-rater reliability and variability of ultrasound measurements of abdominal muscles and fasciae thickness |
| Pizzoferrato 2019 | Perineal ultrasound for the measurement of urethral  mobility: a study of inter- and intra-observer reliability |
| Qureshi 2019 | Anterior segment ultrasound biomicroscopy image analysis using ImageJ software: Intra-observer repeatability and inter-observer agreement |
| Rampinelli 2019 | Inter-observer agreement on the morphology of screening-detected lung cancer: beyond pulmonary nodules and masses |
| Rasmussen 2019 | Inter-rater agreement in the diagnosis of adenomyosis by 2- and 3-dimensional transvaginal ultrasonography Journal of ultrasound in medicine : official journal of the American Institute of Ultrasound in Medicine |
| Rasmussen 2019 | Intra- and Inter-Rater Agreement Describing Myometrial Lesions Using Morphologic Uterus Sonographic Assessment: A Pilot Study Journal of ultrasound in medicine : official journal of the American Institute of Ultrasound in Medicine |
| Razek 2019 | Inter-observer agreement of color duplex ultrasound of central vein stenosis in hemodialysis patients |
| Roach 2019 | Multi-observer contouring of male pelvic anatomy: Highly variable agreement across conventional and emerging structures of interest |
| Rodriguez-Reyna 2019 | Can nailfold videocapillaroscopy images be interpreted reliably by different observers? Results of an inter-reader and intra-reader exercise among rheumatologists with different experience in this field |
| Sevim 2019 | How high is the inter-observer reproducibility in the LIRADS reporting system? |
| Takasaki 2019 | New endoscopic ultrasonography criteria for malignant lymphadenopathy based on inter-rater agreement |
| Tominaga 2019 | Inter-observer agreement in identifying traction bronchiectasis on computed tomography: its improvement with the use of the additional criteria for chronic fibrosing interstitial pneumonia |
| Toriihara 2019 | Comparison of three interpretation criteria of <sup>68</sup>Ga-PSMA PET based on inter-and intra-reader agreement |
| Traverso 2019 | Sensitivity of radiomic features to inter-observer variability and image pre-processing in Apparent Diffusion Coefficient (ADC) maps of cervix cancer patients |
| Trignani 2019 | Inter-observer variability of clinical target volume delineation in definitive radiotherapy of neck lymph node metastases from unknown primary A cooperative study of the Italian Association of Radiotherapy and Clinical Oncology (AIRO) Head and Neck Group |
| Tsai 2019 | Magnetic resonance enterography features of small bowel Crohn's disease activity: An inter-rater reliability study of small bowel active inflammation in clinical practice setting |
| van Ark 2019 | Inter- and intra-rater reliability of ultrasound tissue characterization (UTC) in patellar tendons |
| van Lunenburg 2019 | Sequence and Observer Variability in Gadoxectic Acid-Enhanced MRI Lesion Measurements in Hepatocellular Carcinoma |
| van Riel 2019 | Observer variability for Lung-RADS categorisation of lung cancer screening CTs: impact on patient management |
| Vidiri 2019 | The role of MRI-derived depth of invasion in staging oral tongue squamous cell carcinoma: inter-reader and radiological-pathological agreement |
| Visser 2019 | Inter-rater agreement in glioma segmentations on longitudinal MRI |
| Yoo2019 | Assessment of liver fibrosis using 2-dimensional shear wave elastography: A prospective study of intra-and inter-observer repeatability and comparison with point shear wave elastography |
| Zabala-Travers 2019 | Display colour scale effects on diagnostic performance and reader agreement in cardiac CT and prostate apparent diffusion coefficient assessment |
| **First Author & year** | **Conference abstract title** |
| Baglin 2019 | A 44 channel suprachoroidal retinal prosthesis: Inter-observer reliability measuring electrode to retina distance Investigative Ophthalmology and Visual Science |
| Brites 2019 | Ultrasound inter-reader reliability of inflammatory findings in patients with polyarthritis Annals of the Rheumatic Diseases |
| Calais 2019 | 68Ga-PSMA-11 PET/CT detects prostate cancerat early biochemical recurrence with superiordetectionrate and reader agreement when compared to 18F-fluciclovine PET/CT in a prospective head-to-head comparative phase 3 study Journal of Urology |
| Chmelevsky 2019 | Evaluation of inter-observer variability in ECGI mapping accuracy of ventricular focal arrhythmias Journal of Electrocardiology |
| Colombo 2019 | Inter-observer repeatability of total bone marrow volume measured from WB-MRI in patients with bone metastases Cancer Imaging |
| Demarsin 2019 | High-speed laryngoscopy: Inter-and intra-rater reliability B-Ent |
| Di Biase 2019 | Inter-observer variability in target delineation for brain metastases in stereotactic radiotherapy Radiotherapy and Oncology |
| Eltawil 2019 | Observer agreement on thrombolysis treatment decision in patients with perfusion deficit detected by CTP European Stroke Journal |
| Ferrazzano 2019 | Inter-rater reliability assessment of the neuroimaging common data elements for MRI in severe pediatric TBI Journal of Neurotrauma |
| Frenette 2019 | Intra-and inter-rater reliability of pelvic floor function in women who received pelvic radiotherapy using ultrasound imaging Female Pelvic Medicine and Reconstructive Surgery |
| Hansen 2019 | Analysis of Inter-observer Variation From Meningioma eContouring Session International Journal of Radiation Oncology Biology Physics |
| Henke 2019 | Novel kV CBCT Imager on Ring Gantry Radiotherapy Unit Permits High Inter-rater Contour Uniformity Radiotherapy and Oncology |
| Huang 2019 | Inter-Observer Variations of the Tumor Bed Delineation for Patients after Breast-Conserving Surgery in Preoperative Magnetic Resonance and Computed Tomography Scan Fusion International Journal of Radiation Oncology Biology Physics |
| Jacob 2019 | Evaluation of inter-observer variation for ct identification of childhood interstitial lung disease American Journal of Respiratory and Critical Care Medicine |
| Lao 2019 | Validation and Inter-Rater Reliability of Two Metrics Used as Predictors of Heart Dose in Patients Treated with Adjuvant Radiotherapy to the Left Breast Radiotherapy and Oncology |
| Lovinfosse 2019 | Observer variability in the assessment of renal <sup>18</sup>F-FDG uptake in kidney transplant recipients Journal of Nuclear Medicine |
| Mair 2019 | Reader reliability and accuracy of acute brain imaging interpretation in the enchanted trial: Effectiveness of specialist training for nonexpert readers European Stroke Journal |
| Maugesten 2019 | Inter-reader reliability and comparison of fluorescence optical imaging enhancement in patients with erosive hand osteoarthritis and rheumatoid arthritis Annals of the Rheumatic Diseases |
| Ordidge 2019 | Inter-observer agreement of the FDG PET CT visual Herder score for the assessment of solitary pulmonary nodules European Journal of Nuclear Medicine and Molecular Imaging |
| Patel 2019 | Inter-Observer Agreement between Endosonographers for the Classification of Pancreatic Cysts within a Prospective Randomized Clinical Trial Gastrointestinal Endoscopy |
| Valdovinos-Garcia 2019 | Inter-Observer Agreement for Analysis of Functional Lumen Imaging Probe Topography (Flip) Topography Gastroenterology |
| White 2019 | Inter-observer variability in rectal target delineation on MRI for MR image-guided radiotherapy Radiotherapy and Oncology |
| Willers 2019 | Inter-reader variation in lung segmentation of functional lung MRI quantification European Respiratory Journal |
| Yuan 2019 | GTV definition agreement in brain metastasis radiosurgery using 15T MRI-sim: a multi-observer study Radiotherapy and Oncology |

Supplementary table 6 – Variability measures reported

| **Variability measures** |
| --- |
| Bland Altman (BA) and limits of agreement (LoA) |
| Coefficient of Variation (CoV) |
| Lin’s Concordance Correlation Coefficient (CCC) |
| Minimum Detectable Change (MDC) |
| Standard Error of Measurement (SEM) |
| Gwet’s AC1 index |
| Kendall’s coefficient of concordance |
| Krippendorff’s alpha |
| **Variability measures for contouring studies** |
| Dice similarity coefficient (DSC) |
| Jaccard Index |
| Hausdorff distance |
| Generalised Conformity Index (CIgen) |
| Relative Volume Error (RVE) |
| Volumetric Similarity coefficient (VS) |
| Concordance Index (CCI) |
| Discordant Index (DCI) |
| Centre of Mass distance (CMD) |
| Distance to Agreement (DTA) |
| Mean Absolute Surface Distance (MASD) |
